# Repurposing Infectious Diseases Vaccines Against Cancer

**DOI:** 10.3389/fonc.2021.688755

**Published:** 2021-05-13

**Authors:** Liese Vandeborne, Pan Pantziarka, An M. T. Van Nuffel, Gauthier Bouche

**Affiliations:** The Anticancer Fund, Strombeek-Bever, Belgium

**Keywords:** drug repurposing, oncology, infectious diseases, preventive vaccines, immunotherapy

## Abstract

Vaccines used to prevent infections have long been known to stimulate immune responses to cancer as illustrated by the approval of the Bacillus Calmette–Guérin (BCG) vaccine to treat bladder cancer since the 1970s. The recent approval of immunotherapies has rejuvenated this research area with reports of anti-tumor responses with existing infectious diseases vaccines used as such, either alone or in combination with immune checkpoint inhibitors. Here, we have reviewed and summarized research activities using approved vaccines to treat cancer. Data supporting a cancer therapeutic use was found for 16 vaccines. For 10 (BCG, diphtheria, tetanus, human papillomavirus, influenza, measles, pneumococcus, smallpox, typhoid and varicella-zoster), clinical trials have been conducted or are ongoing. Within the remaining 6, preclinical evidence supports further evaluation of the rotavirus, yellow fever and pertussis vaccine in carefully designed clinical trials. The mechanistic evidence for the cholera vaccine, combined with the observational data in colorectal cancer, is also supportive of clinical translation. There is limited data for the hepatitis B and mumps vaccine (without measles vaccine). Four findings are worth highlighting: the superiority of intravesical typhoid vaccine instillations over BCG in a preclinical bladder cancer model, which is now the subject of a phase I trial; the perioperative use of the influenza vaccine to limit and prevent the natural killer cell dysfunction induced by cancer surgery; objective responses following intratumoral injections of measles vaccine in cutaneous T-cell lymphoma; objective responses induced by human papillomavirus vaccine in cutaneous squamous cell carcinoma. All vaccines are intended to induce or improve an anti-tumor (immune) response. In addition to the biological and immunological mechanisms that vary between vaccines, the mode of administration and sequence with other (immuno-)therapies warrant more attention in future research.

## Introduction

Drug repurposing, which seeks new medical uses of existing licensed drugs, is an active field in cancer research (1, 2). With the growing immunotherapy armamentarium against multiple cancer types, massive research efforts are directed to finding new agents that will further expand the efficacy of these immunotherapies (3, 4). Another proposed strategy is to use existing drugs as add-on to immunotherapy, which represents an affordable and potentially faster strategy that may mitigate the high prices of recent cancer drugs, like immunotherapies (5, 6).

Vaccines used to prevent infections have long been known to stimulate immune responses to cancer, which culminated with the approval of the Bacillus Calmette–Guérin (BCG) vaccine to treat bladder cancer (7). However, BCG has been the exception rather than the rule and the lack of tumor-specific effects of this approach has led cancer vaccine researchers to explore alternative approaches, including the use of existing vaccines as initial vectors to develop tumor-specific vaccines (8).

Recently, several cancer research groups have reported that existing infectious diseases vaccines used as such, either systematically (9) or intratumorally (10–12) or both (13), were able to induce anti-tumor responses, alone or in combination with approved immune checkpoint inhibitors. These experiments were performed in mice, with the exception of a case report (13). While the use of infectious disease vaccines against cancer is not new, the recent approval of immunotherapies has rejuvenated this research area. This has prompted us to survey past and present research activities in the use of infectious diseases vaccines in the treatment of cancer.

Here, we sought to identify repurposing opportunities in the use of infectious diseases vaccines in cancer, focusing on the use of vaccines ‘as is’. Any modification, reformulation or manipulation of an existing vaccine (or drug), though possibly interesting to secure patent protection, renders the clinical development of the ‘new’ product less straightforward (14).

## Methods

In an effort to define a set of vaccine-related search terms for queries on PubMed and clinicaltrials.gov, we identified infectious diseases for which at least one vaccine is approved by the Food and Drug Administration (15) or European Medicines Agency (16), or listed by the World Health Organization (17). This resulted in a list of 31 diseases (**Table 1**) for which more than 80 individual vaccines are marketed.

**Table 1:** List of infectious diseases for which at least one vaccine is available according to the Food and Drug Administration (FDA), European Medicines Agency (EMA) and/or World Health Organization (WHO).

Disease (or micro-organism)	FDA	EMA	WHO
Adenovirus (Type 4 and Type 7)	X		
Anthrax	X		
Cholera	X	X	X
Dengue	X	X	X
Diphtheria	X	X	X
Ebola Zaire	X	X	
Hemophilus influenzae type b	X	X	X
Hepatitis A	X	X	X
Hepatitis B	X	X	X
Hepatitis E			X
Human Papillomavirus	X	X	X
Influenza	X	X	X
Japanese encephalitis	X	X	X
Malaria		X	X
Measles	X	X	X
Meningococcus	X	X	X
Mumps	X	X	X
Pertussis	X	X	X
Bubonic plague	X		
Pneumococcus	X	X	X
Poliomyelitis	X	X	X
Rabies	X	X	X
Rotavirus	X	X	X
Rubella	X	X	X
Smallpox	X	X	
Tetanus	X	X	X
Tick-borne encephalitis			X
Tuberculosis (BCG)	X	X	X
Typhoid	X	X	X
Varicella-Zoster virus	X	X	X
Yellow Fever	X	X	X

We used our methodology, published elsewhere, to interrogate PubMed and clinicaltrials.gov (18). Briefly, a dataset of abstracts and other publication meta-data from PubMed was generated by taking the names of the infectious disease in **Table 1** plus the word ‘vaccine’ (e.g. “influenza vaccine”) conjugated with “cancer” as search terms to generate the PubMed query. We restricted the search to papers published from 1 Jan 2000 until the date of the query performed on 29 October 2020 to focus only on relatively recent articles. For three vaccines that are known to be related to cancer, search terms were modified further in order to minimize irrelevant hits. The first was the BCG vaccine, as it is approved in oncology for the treatment of bladder cancer. The following search terms were used: (“BCG vaccine” OR “BCG vaccination” OR “Bacillus Calmette-Guerin Vaccine” OR “Bacillus Calmette-Guerin Vaccination”) AND cancer NOT (bladder cancer). Next were human papillomavirus (HPV) and hepatitis B virus (HBV) as they are etiologically involved in the carcinogenesis of some cancers. The following queries were used: (“HPV Vaccine” OR “HPV Vaccination” OR “Human papillomavirus vaccine” OR “Human papillomavirus vaccination”) AND cancer NOT (prevention) NOT (cervical cancer); (“HBV Vaccine” OR “HBV Vaccination” OR “Hepatitis B vaccine” OR “Hepatitis B vaccination”) AND cancer NOT (hepatocellular carcinoma).

The PubMed queries were electronically submitted to the Entrez system and the results downloaded and merged into a single dataset for off-line processing. We identified relevant clinical trials on clinicaltrials.gov using the same methodology (download followed by offline manual assessment for relevance; query performed on 3 November 2020). A total of 1754 PubMed abstracts and 549 trial registrations on 28 different vaccines were captured with the search (including duplicates referring to more than one infectious disease vaccine).

Each downloaded abstract and trial was then manually assessed as to whether it was relevant and if so the type of evidence was indicated (e.g. *in vitro*, *in vivo*, case report etc.). Reasons for an abstract or trial to be deemed irrelevant included use of the vaccine for its original indication in cancer patients, use of the vaccine as a vector for developing a new cancer vaccine or use of the vaccine unrelated to oncology. Abstracts were deemed relevant if they reported activity against cancer (positive or negative) – for example, active *in vitro* or *in vivo*, or clinical data showing positive effects on patients treated with the vaccine in question. After initial screening, 164 abstracts and 49 trials were deemed relevant.

**Figure 1:**
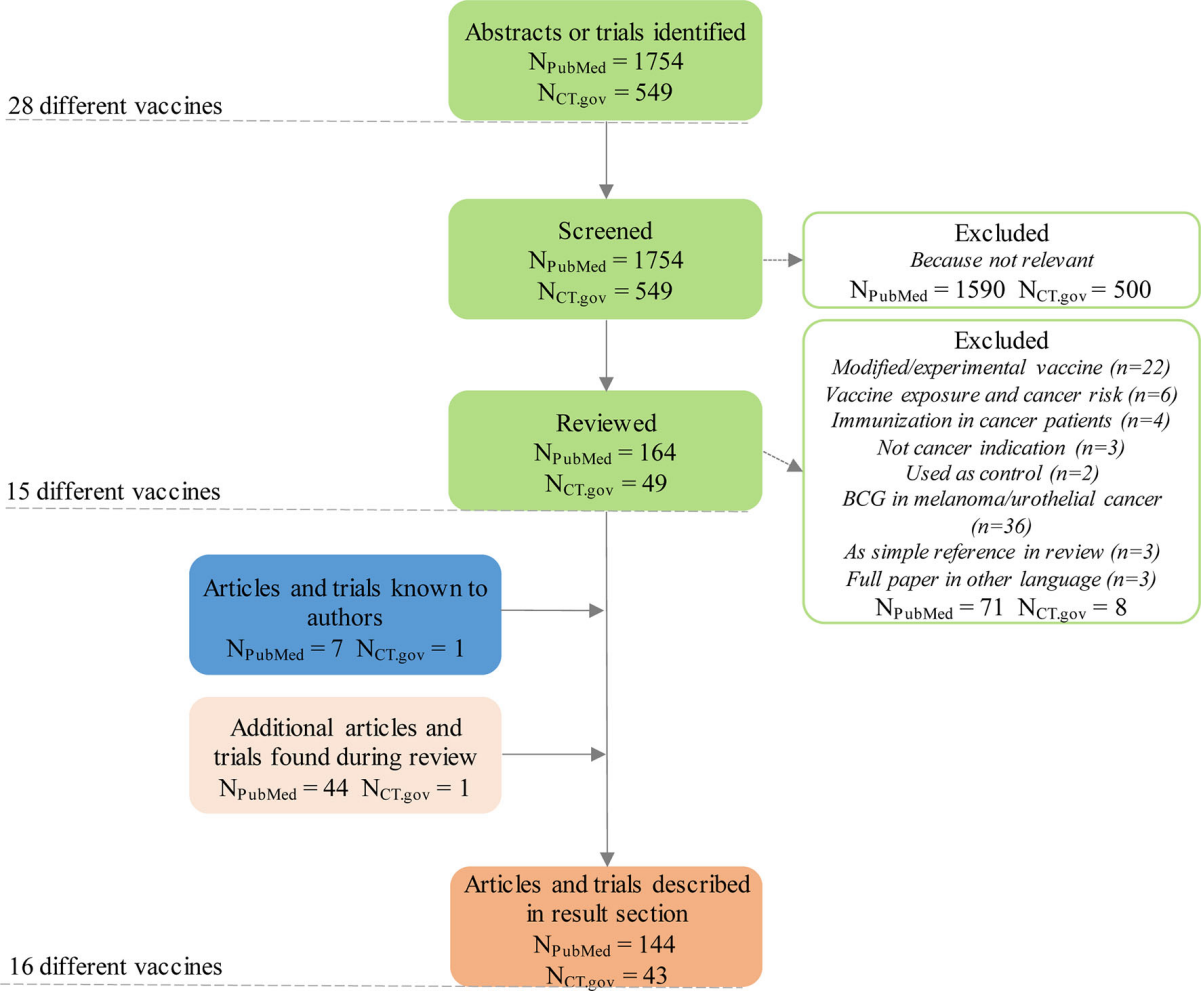
Flow chart of the selection of relevant hits identified through the different queries. N_PubMed_ = articles identified through PubMed database query; N_CT.gov_ = clinical trials identified through clinicaltrials.gov database query.

In a final step, we read all relevant articles and trial registrations. After in-depth review, another 71 abstracts and 8 trials were excluded because of lack of relevance (**Figure 1**). We complemented the list of relevant abstracts and trials with articles not captured by the search but known by any of the co-authors. The rotavirus vaccine, for example, was added as one article known to one of the co-authors had not been captured by the search (11). We then summarized the main findings of the remaining articles and trial registrations, complementing them with additional references cited in or referring to articles captured by our search. Characteristics of these 16 vaccines with anticancer evidence, and main features from literature are summarized in **Table 2**. Summaries of results are described for each vaccine below (in alphabetical order).

**Table 2:** Overview of literature findings and proposed next steps for vaccines with repurposing potential in oncology.

Vaccine	Type	Trials	Summary of the main literature findings	Proposed next steps
BCG vaccine	Live attenuated bacterium	15 registered but many trials preceded trial registries	BCG has been extensively used as an adjuvant to cancer vaccines (autologous cancer cells or dendritic cells-based) without any clinical success so far. Intratumoral BCG is listed in melanoma guidelines and is used in spontaneous cancers in large animals. This supports combining BCG with other drugs in specific cancer types. However, the rather nonspecific immune stimulation induced by BCG may make BCG less attractive than other intratumoral immunomodulators.	Early-phase clinical trials combining intratumoral BCG injections with approved therapies.
Cholera vaccine	Inactivated/killed bacterium	None	Three positive pharmaco-epidemiologic studies in colorectal, prostate, and breast cancers. ‘Desirable’ immune and inflammatory changes induced by the vaccine observed in the colon in animal models.	Preclinical work in colorectal cancer models to further characterize efficacy and mode of action. If positive, preclinical work could extend to prostate and breast cancer models as well.
Diphtheria vaccine	Toxoid (inactivated bacterial toxin)	NCT03299309, NCT03927222, NCT03688178, NCT02193347, NCT03615404, NCT02366728, NCT02465268, NCT02338700, NCT02338752, NCT02338778, NCT02338804, NCT02333474	The Td vaccine has been used to augment anti-tumor immune responses expected from experimental immunotherapies, with various proposed mechanisms. The first 7 trials listed use Td as a preconditioning strategy to augment the effect of experimental immunotherapies (either peptide vaccines or cell-based therapies) in patients with brain cancer. The other 5 trials (no results published) combine the diphtheria vaccine with other infectious disease vaccines in patients with prostate, pancreas, liver, breast or lung cancer.	Currently no next steps identified because awaiting trial results in brain, prostate, pancreas, liver, breast and lung cancer.
Hepatitis B vaccine	Inactivated virus/recombinant DNA	None	Limited evidence summarized in a review mainly building on the use of HBV antigens in cancer experiments.	Confirmation of a potentiating role of HBV vaccine with approved immunotherapies in preclinical models.
Human Papillomavirus vaccine	Viral subunit/conjugate	NCT03051516, NCT04096911	Systemic responses observed in patients with SCC of the skin injected intratumorally and/or intramuscularly with HPV vaccines. The trials listed explore if HPV vaccine can prevent the recurrence of pre-cancerous anal or vulvar lesions, and, in combination with anti-PD1, treat incurable HPV+ cervical cancer.	In cutaneous SCC, an efficacy phase 2 trial might be an appropriate next step. With regards to HPV+ cervical cancer, results of the ongoing trial are to be awaited.
Influenza vaccine	Inactivated/killed virus, viral subunit/conjugate	NCT02998736, NCT04591379	The ability of the vaccine to restore NK cell dysfunction induced by cancer surgery, shown in animal models of lung metastases, is being tested in patients with abdominal malignancies in the trial listed. A pilot trial of intratumoral injection before colorectal cancer surgery is also ongoing. Additional *in vivo* evidence for a role of intratumoral injection to improve anti-PD-1 efficacy in a murine melanoma model exists.	For peri-operative use in colorectal cancer or lung metastases, results of ongoing trials are awaited. Efficacy of intratumoral influenza vaccine in combination with immune checkpoint inhibitors could be investigated in an early phase trial involving melanoma and/or triple negative breast cancer patients.
Measles vaccine	Live attenuated virus	NCT00828022, (19)	Vaccine has been shown to be cytotoxic in multiple cancer cell lines with *in vivo* confirmation, most often (but not always) after intratumoral injection, in models of myeloma, lymphoma, leukemia, laryngeal, lung, melanoma, ovarian and prostate carcinoma. A trial of measles vaccine as consolidation therapy in lung cancer has not yet reported results, whereas a phase I trial of intratumoral injection in cutaneous T-cell lymphoma patients has shown its ability to induce tumor regression.	Research strategy should be streamlined as research is currently going in multiple directions. Additional preclinical work and/or a phase I trial in an injectable tumor for which *in vivo* data exist, could be relevant, in addition to a phase 2 trial in T-cell lymphoma. Depending on the outcome of the phase 1/2 lung cancer trial, this strategy could also be further explored.
Mumps vaccine	Live attenuated virus	None	Supporting data in animal models of AML, ovarian and prostate cancer.	Additional preclinical work in ovarian cancer, perhaps in combination with the measles vaccine.
Pertussis vaccine	Bacterial subunit/conjugate Inactivated/killed bacterium	None	A case report of a dramatic response in a melanoma patient after diphtheria-tetanus-pertussis vaccination. The pertussis vaccine has been used as an adjuvant to improve anti-tumor immune responses in animal models of lymphoma.	Preclinical work on the diphtheria-tetanus-pertussis vaccine in melanoma models and on the whole-cell pertussis vaccine in lymphoma.
Pneumococcus vaccine	Bacterial subunit/conjugate	(20), NCT01351896, NCT03376477, NCT03942328	All ongoing and reported trials use the pneumococcus vaccine in combination with lenalidomide or experimental immunotherapies (cancer vaccines or autologous dendritic cells) to help eliciting anti-tumor responses in patients with chronic lymphocytic leukemia, lymphocytic lymphoma, multiple myeloma and liver cancer.	Currently none as trials are ongoing in chronic lymphocytic leukemia, lymphocytic lymphoma, multiple myeloma and liver cancer. Results to be awaited.
Rotavirus vaccine	Live attenuated virus	None	In a series of animal experiments, intratumoral injections of the rotavirus vaccine could overcome resistance to checkpoint inhibitors in 4 different models (neuroblastoma, lymphoma, breast and colon carcinoma) and strongly synergize with checkpoint inhibitors in a neuroblastoma and a lymphoma model.	A proof-of-concept trial in neuroblastoma and lymphoma is necessary.
Smallpox vaccine	Live attenuated virus	NCT04410874	A positive observational study in melanoma patients. Attempts to vaccinate melanoma patients with a melanoma-vaccinia lysate have failed in randomized clinical trials. Canadian phase 1/2 trial is currently recruiting patients with basal cell carcinoma and squamous cell carcinoma for intratumoral treatment with a smallpox vaccine.	Currently none as trial in non-melanoma skin cancer is ongoing.
Tetanus vaccine	Toxoid (inactivated bacterial toxin)	(21, 22), NCT02366728, NCT02737475	The Td vaccine has been used to augment anti-tumor immune responses expected from experimental immunotherapies, with various proposed mechanisms. One trial in colorectal cancer did not show any role for Td when added to an experimental cancer vaccine. One trial in glioblastoma suggests that priming DC with Td is beneficial. This led to an ongoing glioblastoma trial. The last trial uses Td with an experimental drug in advanced cancers (but no rationale is available).	Cf. Diphtheria vaccine
Typhoid vaccine	Live attenuated bacterium, bacterial subunit/conjugate	NCT03421236 (23),	Based on preclinical data in a mice model of bladder cancer showing an effect superior to the BCG vaccine, a phase I trial of intravesical instillations has been initiated in bladder cancer patients. DC therapy generated using a mix of BCG vaccine, typhoid vaccine and a vaccine against *Haemophilus influenzae*, plus prostaglandin E_2_ showed improved survival in stage IV melanoma patients.	Currently none as bladder cancer trial is ongoing.
Varicella vaccine	Live attenuated virus	NCT01953900	The VZV vaccine is used to expand experimental T-cells targeting GD-2 in a trial protocol in osteosarcoma and neuroblastoma.	Currently none as trial in neuroblastoma and osteosarcoma with the vaccine and anti-GD2 T-cell is ongoing.
Yellow Fever vaccine	Live attenuated virus	None	Mixed results from observational study in melanoma patients. Preclinical data supporting a role for intratumoral injection of the yellow fever vaccine in animal models of colon cancer and melanoma.	More preclinical work with animal models and a phase I trial in melanoma based on observational study and preclinical data.

BCG, Bacillus Calmette–Guérin; Td, tetanus diphtheria; HBV, hepatitis B virus; HPV, human papillomavirus; SCC, squamous cell carcinoma; NK, natural killer; PD-1, programmed cell death protein 1; AML, acute myeloid leukemia; DC, dendritic cell; VZV, varicella-zoster virus; GD-2, disialoganglioside.

## BCG Vaccine

BCG contains live, attenuated *Mycobacterium bovis* and is since the 1920s the only available tuberculosis vaccine. BCG is also approved and recommended for the treatment of non-muscle invasive bladder cancer. This approval is the end result of a clinical development conducted in the 1970s and 1980s in multiple cancer types. This program has led to mixed results with successes in non-muscle invasive bladder cancers and melanoma, but discontinuation or failure in other tumor types, including in phase 3 trials for ovarian and small-cell lung cancer (24). Since our report focuses on publications after 2000, a few important points need to be made before discussing the abstracts identified by our methodology.

1) The literature on BCG and non-urological cancers has been reviewed in 2020 by Noguera-Ortega et al. Their comprehensive review is a very useful tool to navigate through the wealth of data published since the 1930s about BCG experiments in non-urological cancer types. It also includes data with mycobacteria other than BCG (24).
2) Preceding its use in bladder cancer, BCG had been extensively studied and consequently recommended for melanoma. BCG is injected intratumorally into melanoma lesions, with cumulative evidence leading to its recommendation for in-transit lesions (25), an option still listed in the 2020 National Comprehensive Cancer Network melanoma guidelines. Since BCG use in melanoma is listed in guidelines and has been extensively reviewed elsewhere (26, 27), it is not covered in our report. According to these reviews, BCG is an important, though neglected, therapeutic tool to be considered for improving outcomes of melanoma patients, in particular in new combinations.
3) Colon cancer is one notable exception to the negative results in non-urological and non-melanoma cancers. Long-term results from a phase 3 trial conducted by the National Surgical Adjuvant Breast and Bowel Project collaborative group showed an improved overall survival with the use of adjuvant BCG after surgery compared to observation but those results have not changed clinical practices (28).
4) Building on the BCG success, multiple mycobacteria-based products have been developed and tested in clinical trials (24). One product called mifamurtide, an analogue of muramyl dipeptide which is a component of *Mycobacterium* cell wall, is approved in Europe since 2009 for the treatment of osteosarcoma after surgical resection (29).

The literature published on BCG and cancer since 2000 is rich. Most of the abstracts identified can be categorized into 3 categories.

### BCG as an Adjuvant to Cancer Vaccines

Twenty relevant articles from our search report either laboratory experiments or clinical trials where the BCG vaccine is used as an adjuvant to an experimental tumor vaccine. This area of research had started years before 2000 and interest in BCG as an adjuvant to tumor vaccines has continued at least until 2012 (date of the last abstract falling in this category). These vaccines mainly consist of either dendritic cells or mixed tumor cells (30–47). Most notably, two phase 3 trials – one in limited-stage small cell lung cancer patients with a Bec2/BCG vaccine and one in stage II-III colon cancer patients with a BCG/autologous tumor cell vaccine – failed to demonstrate any benefit over observation (31, 32). Twelve registered trials using BCG as an adjuvant to an experimental cancer vaccine were identified (NCT00514072, NCT00003279, NCT00006352, NCT00003184, NCT00037713, NCT00007826, NCT00671554, NCT00003023, NCT00016133, NCT00427570, NCT00003386, NCT00075569, NCT00003715, NCT01729663). All are completed or terminated and none of the products tested has been approved.

### BCG as Direct Anticancer Therapy (Alone or in Combination With Existing Treatments)

Clinically, we found data on intra-pleural use of BCG in patients with small cell lung cancer and non–small cell lung cancer (NSCLC), however with no success so far [reviewed in (48, 49)]. These failures have been attributed to the nonspecific immune stimulation induced by BCG. One patient with hepatocellular carcinoma was treated with a combination of interleukin-2 (IL-2) injection, BCG and melatonin. Alpha-fetoprotein level went down from 5,000 IU/ml to undetectable with 6 out of 7 tumors regressing on the first follow-up scan (50). We also identified 2 recently registered trials using intralesional BCG in melanoma. One trial combined intralesional BCG with intravenous ipilimumab, an antibody targeting CTLA-4, but the trial was stopped early as two of five patients developed high-grade immune-related adverse events, which was attributed to the combination (51). Another melanoma trial, not yet recruiting, will compare response rates between patients receiving intralesional IL-2 alone and patients receiving intralesional IL-2 and intralesional BCG (NCT03928275).

BCG has also been used to treat cancers in bovines, equines and dogs, alone or in various combinations. In one study, six animals with localized bovine ocular squamous cell carcinoma were treated with four BCG intralesional injections given over eight weeks. All animals recovered completely with no recurrence at one year (52). Three papers report on BCG use in the treatment of sarcoids in equids (53–55). BCG is one of the therapeutic modalities deemed effective. In the largest study, 28 of 48 sarcoid tumors (58%) regressed completely with no recurrence for at least 6 months (55). In a randomized clinical trial in dogs with canine mast cell tumors, dogs treated with subcutaneous injections of BCG and human chorionic gonadotropin had a response rate (28%) similar to that of dogs treated with vinblastine, but experienced less neutropenia (56). In another canine trial, this time in dogs with transmissible venereal tumors, 20 dogs were divided in four equal groups and treated with intratumoral saline (controls), intratumoral BCG, intravenous vincristine (vinca alkaloid chemotherapeutic agent) or both intratumoral BCG and intravenous vincristine. All groups except the control group experienced some level of tumor regression, with dogs in the combination group experiencing the largest and fastest reduction of tumor size. Histologically, BCG induced major infiltration of T-cells and macrophages in the tumor (57).

Most experiments with BCG published since 2000 have been conducted to further characterize the immune effect of BCG with modern research tools. Out of nine patients with various solid tumors injected with intratumoral BCG and concomitantly treated with systemic cyclophosphamide (chemotherapeutic and immunosuppressive drug), two experienced a partial response (one breast cancer and one uterine sarcoma patient). Histological analyses of five of the nine patients showed a major infiltration of the tumor by myeloid cells, CD11c+ immature dendritic cells (DC), CD68+ macrophage cells and CD8+ T cells (58). In mice experiments using B16 melanoma and AKR lymphoma models, Kaptzan et al. looked at the effect of intratumoral BCG injections on tumor growth and survival in both young and old mice. BCG reduced tumor growth and prolonged survival only in old mice which was attributed to a more pronounced infiltration of T-cells and macrophages in tumors of old mice (59).

Several articles have looked at the detailed effect of BCG on cancer cell lines and on human leukocytes *in vitro*, including two papers using leukocytes from head and neck cancer patients (60–63). Overall, BCG increased production of most cytokines measured but most notably IL-6, tumor necrosis factor and interferon-gamma, but not IL-2. BCG increased cytotoxicity in head and neck and in gastric cancer cell lines (62, 63). Liu et al. showed that BCG increased human leukocyte antigen 1 expression in three of the five tumor cell lines tested. In the same three cell lines, CD8+ T-cells cytotoxicity was enhanced in BCG-treated cells (61). In another paper from the same group, the immunotherapeutic effect of BCG was attributed to γδ T-cells’ anti-tumor response induced by cytokines from type-1 dendritic cells (64). In a leukemia mouse model, Basu et al. have shown that BCG could increase survival thanks to its stimulating effect on hematopoietic regeneration (65). In a VX-2 squamous cell carcinoma rabbit model in which rabbits are injected with tumor cells on two sites (auricle and lung), Hamamoto et al. looked at the effect of intralesional BCG on radio-frequency ablation of the lung tumor. Both radio-frequency ablation alone and the radio-frequency ablation combined with BCG increased survival compared to controls but only the combination was able to reduce the size of the second tumor in the auricle, indicative of a systemic effect of the local combination (66).

### Increased Risk of Malignancy Following BCG Injection

Several papers report the development of various forms of benign, borderline and malignant tumors at the site of BCG injections. In some cases, basal cell or squamous cell carcinomas of the skin have developed on a BCG scar or granuloma decades after childhood BCG vaccination (67–70). In other cases, and more relevant to the possible therapeutic use of BCG vaccines, skin squamous cell carcinoma (71, 72) or tufted angioma (73) arose weeks or months after BCG vaccination. In one patient with pre-existing Kaposi form hemangioendothelioma, a Kasabach-Merritt phenomenon was reported just after BCG vaccination (74). A second case is described in the same report, this time following tetanus-diphtheria-pertussis vaccine, suggesting this is not specific to BCG. In one other case report, a patient with osteosarcoma received a BCG vaccination as an immunostimulant prior to surgery and subsequently developed cutaneous tuberculosis (75).

Overall, BCG may have potential in cancer types other than urological cancers. In melanoma, clinical adoption has remained limited for years and with the approvals of BRAF/MEK inhibitors and immune checkpoint blockers, the role of BCG is now even less clear. Using BCG as an adjuvant to cancer vaccines (autologous cancer cells or dendritic cells-based) has faded over time, at least in part because of the repeated clinical failures of the experimental vaccines tested. The ability for intratumoral BCG to recruit immune cells in the tumor has been well documented since 2000. This body of research, which includes data in human melanoma and spontaneous cancer in large animals, is supportive of combining BCG with other drugs in specific cancer types. However, the rather nonspecific immune stimulation induced by BCG may make BCG less attractive than other intratumoral immunomodulators.

## Cholera Vaccine

Prophylactic oral cholera vaccines, containing inactivated *Vibrio cholerae* bacteria, are given to travelers and people living in countries at high risk of infection since the 1990s. The evidence supporting the investigation of the cholera vaccine in oncology comes mainly from three pharmaco-epidemiology studies reported by Ji et al. in 2017, 2018 and 2020. Linking data from the Swedish Cancer Register and the Swedish prescribed drug register, they evaluated the association between the use of the cholera vaccine and death from colorectal cancer, death from prostate cancer and death from breast cancer. In the colorectal cancer population, having received the cholera vaccine was associated with lower colorectal cancer mortality (HR, 0.53; 95% CI, 0.29–0.99) and all-cause mortality (HR, 0.59; 95% CI, 0.37–0.94). In the prostate cancer population, a similar association was reported for both prostate cancer mortality (HR, 0.57; 95% CI, 0.40–0.82) and all-cause mortality (HR, 0.53; 95% CI, 0.41–0.69). Finally, in breast cancer patients, associations were also significant for both breast cancer mortality (HR, 0.53; 95% CI, 0.33–0.84) and all-cause mortality (HR, 0.54; 95% CI, 0.37–0.79). Special care was taken to account for confounding, indication bias and immortal-time bias. These associations were maintained in complementary analyses exploring these biases, though one cannot definitely rule out residual confounding or other possible biases (76–78).

Preclinically, the cholera toxin showed an anti-inflammatory effect on the colon (79). Of particular relevance, oral administration of a low non-pathogenic dose of the cholera toxin reduces colonic polypoid genesis in mice (80). It is suggested that the cholera toxin B subunit, which is part of the approved oral vaccines, is responsible for these immune and inflammatory changes (81).

## Diphtheria Vaccine

Diphtheria vaccines containing inactivated toxins have been used for decades as they are highly effective in preventing infection with *Corynebacterium diphtheriae*. The diphtheria toxoid is also available in combination with other toxins and antigens. Though no relevant abstracts were identified by our methodology with the search term “diphtheria vaccine”, we did find several trial registrations. Most are related to tetanus-diphtheria toxoids (Td) used as preconditioning regimen for the enhancement of experimental anticancer treatments’ efficacy. A case report of a melanoma regression with the trivalent diphtheria-tetanus-pertussis vaccine is described in the pertussis section (82).

At Duke University Medical Center, six trials in brain tumors (gliomas and medulloblastomas) with Td as preconditioning vaccine in combination with various experimental immunotherapies were registered: as of March 2021, three were recruiting patients (NCT03299309, NCT03927222, NCT03688178), two were active, not recruiting (NCT02193347, NCT02366728) and one was completed with no results available (NCT03615404).

Similar to trials sponsored by Duke University, the phase 2 trial (ATTAC-II - NCT02465268) led by Dr. Rahman’s team at the University of Florida is recruiting newly diagnosed glioblastoma patients to investigate if a cytomegalovirus pp65-mRNA-pulsed DC vaccine is effective when given with stronger doses of routine chemotherapy and preconditioning with Td.

The Chinese team of Ke-Cheng et al. ran five phase 1/2 trials (NCT02338700, NCT02338752, NCT02338778, NCT02338804 and NCT02333474) evaluating safety and effectiveness of a mixed vaccine (consisting of diphtheria, pertussis, tetanus, typhoid, Staphylococcus aureus and paratyphoid A and B) combined with standard treatment for patients diagnosed with prostate, pancreatic, hepatocyte, breast and lung carcinoma, respectively. Although trials are marked as completed on clinicaltrials.gov, no data is published to date.

## HBV Vaccine

Inactivated HBV vaccines are used to prevent hepatitis B viral infection. The recombinant vaccine composed of the hepatitis B virus “s” antigens (part of the outer envelope of the virus) are on the market since the 1980s. In a comprehensive review, Altinoz et al. discuss the role of the hepatitis B vaccine to prevent and treat cancer. They report evidence for the use of HBV antigens in combination with experimental therapies both in clinical and preclinical studies. Though none of the studies reported has used existing vaccines as such, Atlinoz et al. strongly argue that the data accumulated on HBV antigens support testing HBV vaccines in combination with immunotherapies in oncology (83).

## HPV Vaccine

By preventing infection by various HPV types, in particular type 16 and 18, HPV vaccines are used to prevent cervical cancer since the 2000s. Today, vaccines composed of viral subunits exist that target either nine (9-valent), four (quadrivalent) or two (bivalent) HPV types. Because HPV infection has also been suggested to play a role in the development of squamous cell carcinomas (SCCs) of the skin (84, 85), Nichols et al. offered three intramuscular doses of the quadrivalent HPV vaccine (Gardasil^®^, MSD) to two immunocompetent patients with a history of multiple keratinocyte carcinomas, without known HPV infection, and observed a reduction in the development of new keratinocyte carcinomas in both patients (86). However, with a small cohort and absence of long-term data, concerns about selection bias exist (87).

These observations led them to use the 9-valent HPV vaccine (Gardasil-9^®^, MSD) in an immunocompetent woman in her late nineties, suffering from multiple inoperable cutaneous basaloid SCCs and refusing systemic chemotherapy. Over a period of 11 months, the patient received two doses of intramuscular HPV vaccine and four doses intratumorally into three of the largest tumors, leading to resolution of all lesions, including the non-injected ones. Until end of follow-up, two years after first intratumoral administration, there was no clinical or histologic evidence of residual SCC (13). Based on this experience, Nichols et al. offered the same treatment to an 87-year-old immunosuppressed renal transplant recipient diagnosed with an inoperable SCC *in situ* on the left hand and refusing radiotherapy. Over four months, the patient received two intramuscular and two intratumoral injections with the 9-valent HPV vaccine (Gardasil-9^®^, MSD). Five months after the last injection, the lesion resolved with biopsy-proven histologic cure (88).

Geizhals and Lebwohl reported the case of an 84-year-old immunocompetent female presenting with multiple invasive cutaneous SCCs on her right leg. After failing initial treatment, they treated her with two intramuscular and three intratumoral injections of the 9-valent HPV vaccine over a 9-month period. Ten months after first injection, there was no clinical or histologic evidence of residual SCC (89).

These anecdotal studies give rise to several questions including whether successful outcomes depend on tumor HPV status, whether some epithelial tumor types may be more responsive than others, what the predictors of response are, and, for reasons of both safety and efficacy, what the mechanism of action is (90, 91).

It should be noted that a possible therapeutic benefit of HPV vaccination has been observed for preventing the recurrence of pre-cancerous lesions as well (91–94). The evidence is compelling for prevention of pre-cancerous cervical lesions in patients who underwent conization for cervical intraepithelial neoplasia (95), but is also growing for other HPV-associated lesions (91, 93, 94). Karita et al., for instance, are running a clinical trial evaluating whether Gardasil-9^®^ (MSD) can reduce the risk of high-grade squamous intraepithelial lesion recurrence by 50% in previously unvaccinated individuals recently treated for anal or vulvar high-grade squamous intraepithelial lesions (NCT03051516) (96).

Besides pre-cancerous lesions, a possible therapeutic effect of HPV vaccination in established HPV-positive tumors is being investigated. Gustafson et al. reported the case of a 67-year-old woman with vulvar SCC who, after three relapses, was treated maximally with radiation therapy and surgery. She was offered Gardasil^®^ vaccination (MSD) with the intention to prevent recurrence. A metastatic lymph node was diagnosed and resected 15 months after vaccination. Almost 24 months later, the patient had not experienced any further relapse (97).

Gardasil^®^ (intramuscular) in combination with Sintilimab (anti-PD1) is being investigated for efficacy in a Chinese phase 2 trial recruiting patients with persistent, recurrent or metastatic HPV+ cervical cancer not amendable to curative therapy (NCT04096911).

In accordance with previous case reports and meta-analysis, a recently published review paper reported that in 26 case control studies, high risk HPVs have been identified in prostate cancers (22.6%) and in normal or benign prostate controls (8.6%) (p = 0.001). Lawson et al. concluded that a causal role for HPVs in prostate cancer is highly likely which suggests that the HPV vaccine may also be relevant for future prostate cancer research (98).

## Influenza Vaccine

Immunization as an effective strategy to prevent influenza virus infection started in the 1930s. As circulating influenza virus strains change constantly, influenza vaccines need to be changed annually. Numerous seasonal influenza vaccines, containing inactivated/killed virus or viral particles of at least 2 influenza A strains and 1 influenza B strain, are available. After demonstrating that surgery causes a general dysfunction in natural killer (NK) cells (99), Tai et al. hypothesized that non-specific stimulation of the immune system (such as an infectious disease vaccination) could prevent postoperative NK cell dysfunction and attenuate the metastatic dissemination of malignant cells if administered before surgery (100). Using murine models of metastasis, and surgical stress, they demonstrated that a single intramuscular dose of influenza vaccine delivered one day before surgery resulted in optimal activation of NK cells and a dramatic reduction in the metastatic dissemination of cancer cells to the lungs (9). Based on these results, a phase 1 clinical trial has been initiated (NCT02998736) to evaluate the safety and the NK cell killing effect of perioperative administration of intramuscular influenza vaccination in combination with the phosphodiesterase-5 inhibitor, tadalafil, to alleviate myeloid derived suppressor cell inhibition of NK cells in patients with abdominal cancer undergoing surgery (101).

Additional supporting evidence for the use of influenza vaccine at the time around cancer surgery came from an observational Danish study. Inactivated trivalent influenza vaccination given within 6 months after curative surgery for solid tumors was associated with a decreased mortality (HR, 0.89; 95% CI, 0.81-0.99). This association seemed to be driven by vaccination within the first 30 post-operative days (HR, 0.73; 95% CI, 0.60-0.89) (102). The investigators have initiated a pilot trial looking at the safety of intratumoral flu vaccine injection prior to curative surgery of colorectal cancers (NCT04591379).

Besides perioperative use, the influenza vaccine has recently been investigated in combination with immunotherapy as well.

Related to immune checkpoint inhibitors, Newman et al. provided preclinical evidence that intratumoral, and not intramuscular, injection of the 2017-2018 seasonal unadjuvanted influenza vaccines approved by the Food and Drug Administration (Flucelvax^®^, Seqirus; Fluvirin^®^, Seqirus; Fluarix^®^, GlaxoSmithKline; Flublok^®^, Protein Sciences Corporation) reduced tumor growth in mice in a melanoma model. Adjuvanted vaccine (Fluad^®^, Seqirus) was only able to reduce tumor growth after removal of the adjuvant. Combination of the unadjuvanted influenza vaccines with PD-L1 checkpoint blockade led to an even greater tumor growth reduction, even in tumors resistant to checkpoint blockade treatment alone. The authors posited that immunologically inactive cold tumors are converted into immune-infiltrated hot tumors due to an increase in DCs, including cross-presenting DCs, and tumor antigen-specific CD8^+^ T cells within the tumor microenvironment (10).

From a clinical standpoint, though initial experience of influenza vaccination in cancer patients concomitantly receiving anti-PD-(L)1 suggested an increased rate of adverse events (103), this safety concern was not confirmed in larger cohorts (104–106). In the INVIDIa study in advanced cancer patients treated with immune checkpoint inhibitors, receiving the flu vaccine and/or developing a flu-like syndrome was actually associated with longer overall survival. No statistically significant differences were seen in terms of overall response rate, disease control rate or time to treatment failure with immune checkpoint inhibitors between vaccinated and control patients (107). A Chinese observational study (NCT04355806) in NSCLC patients receiving anti-PD-(L)1 treatment and a seasonal intramuscular injection of trivalent influenza vaccine, is ongoing. Besides immunogenicity and safety, oncological outcomes such as overall survival and progression-free survival will be assessed.

Related to cell-based immunotherapy, preclinical and clinical research on the influenza vaccine used in combination with other vaccines for DC maturation is described in the typhoid section.

Based on data from Medicare claims between 2013 and 2017, Pachynski et al. did not report an impact of concurrent use of the flu vaccine on survival outcomes after sipuleucel-T administration, an autologous cellular immunotherapy, in men with metastatic castration resistant prostate cancer (108).

It should be noted that Edwards-Bennet et al. reported a rare case of a 67-year-old female who developed mucosa-associated lymphoid tissue lymphoma of the right triceps muscle after an influenza vaccination at the same site one month earlier. Whether the mucosa-associated lymphoid tissue was coincidental or related to vaccination remains undetermined (109).

## Measles Vaccine

Since the 1960s, the measles vaccine is used effectively in prevention of measles disease. Live, attenuated virus makes up the vaccine by itself and the combination vaccines such as the MMR vaccine (a combination with the rubella vaccine and mumps vaccine) or the MMRV vaccine (a combination of MMR with the chickenpox vaccine). The oncolytic activity of the measles virus (MeV) is one of the most systematically studied among infectious disease viruses (110). From the 1970s onwards, a number of case reports have been published describing remissions of Burkitt’s lymphoma (111) and Hodgkin’s disease (112, 113) under MeV infection.

In terms of mechanism of action, two receptors for MeV have been identified: signaling lymphocyte activation molecule (SLAM) and CD46. The role of both receptors in MeV-mediated pathology is unclear. However, for cell entry, the wild type MeV mostly relies on SLAM, while the live attenuated vaccine strains of MeV use CD46 receptor (114, 115). Next to the immune stimulating effect, the cytopathic effect of MeV is characterized by the formation of syncytia (giant multinucleated cells) due to the interactions of viral hemagglutinin and fusion proteins from the infected cells with the CD46 expressed by the neighboring cells (116). Both receptors play a role here; SLAM seems important for T-cell activation and CD46 would account for the preferential killing of tumor cells due to its abundant expression on transformed cells (114, 115).

Research involving genetically re-engineered MeV is ongoing, aiming at increasing the efficiency of anti-tumor virotherapy. Recombinant viruses expressing the sodium iodide symporter, carcinoembryonic antigen, colony stimulating factor, suicide genes, etc. are in development (117–119).

The attenuated MeV has been used safely across the world in pediatric vaccination programs for many years. This makes vaccine strains of MeV very attractive repurposing candidates (116, 120). Because of the many articles about the use of the measles vaccine for cancer, we report the evidence per cancer type in alphabetical order.

### Blood Cancers

Almost 20 years ago, Peng et al. demonstrated strong cytopathic effects of an attenuated Edmonston-B vaccine strain of MeV in myeloma cell lines. In a murine xenograft model for myeloma, tumor growth in animals treated with intratumoral MeV-injection was significantly inhibited (P = .002) compared to control group. Survival of mice repeatedly treated with intravenous MeV was considerably longer than the control group. Complete tumor regression was achieved in one of the eight treated mice implanted with RPMI-8226 myeloma cells and in six of six treated mice implanted with ARH-77 myeloma cells (121).

Grote et al. also reported that intratumoral injection of the attenuated Edmonston-B vaccine strain of MeV induced regression of large established human B-cell lymphoma xenografts in severe combined immunodeficient mice whereas all tumors in the control group treated with UV-inactivated virus progressed. Intravenous administration of MeV (daily for 10 days) also considerably slowed down tumor progression (122).

Zhang et al. showed that an increased induction of apoptosis is responsible for the cytotoxic effect of the combination of vaccine-derived MeV (Schwarz strain) and mumps virus on hematopoietic cancer cell lines (acute myeloid leukemia (AML), T-cell leukemia, T-lymphoma and B-lymphoma lines). Though not statistically significant, the authors observed in an AML xenograft model, that intratumoral injections (twice a week for three weeks) of both the measles and mumps virus showed slower tumor development and prolonged survival compared to single-virus treated and control mice. *Ex vivo* evaluation on AML patient samples showed that the combination of both viruses efficiently killed blasts in 16 of 20 patient samples. Greater cell killing was observed after administration of the viruses plus the chemotherapeutic agent cytarabine in 11 out of these 16 samples compared to either treatment alone. However, in 4/20 samples, blast cell growth was only maintained upon infection with both viruses, raising the possibility that the viruses may stimulate cell growth, underlining the need for careful validation and in-depth characterization of resistance (123).

Heinzerling et al. evaluated the effect of MeV in cutaneous T-cell lymphomas in a phase 1 dose escalation trial. After pre-treatment with interferon-α to prevent uncontrolled virus spread, five patients with clinically measurable disease and anti-MeV antibodies prior to study inclusion, were injected with the MeV vaccine (purified from MMR^®^ vaccine, Berna Biotech) intratumorally. Treatment was well-tolerated and resulted in clinical responses. Five of the six lesions injected (one patient had two lesions injected) showed regression. Distant non-injected lesions improved in two patients but remained unchanged in the other three patents. Overall, best response was stable disease in two, partial response in one and progressive disease in two patients (19).

### Breast Cancer

Abdullah et al. evaluated the oncolytic effect of a live attenuated MeV vaccine (Serum Institute of India PVT. Ltd.) in an Iraqi patient-derived breast cancer cell line (AMJ13) and two international breast cancer cell lines (MCF-7 and CAL-51). The MeV infected and destroyed breast cancer cells, especially the AMJ13 cells: after 72 h of infection, the proportion of AMJ13 apoptotic cells was 83%, while for MCF-7 and CAL-51 were 70% and 67% respectively (124).

### Laryngeal Cancer

Toan et al. assessed the anticancer effects of MeV (purified from Priorix^®^, GSK) in combination with nimotuzumab, a monoclonal antibody targeting the epidermal growth factor receptor, *in vitro* and *in vivo*. Hep2 laryngeal cancer cells treated with MeV and nimotuzumab had a significantly lower survival rate compared to those treated with MeV or nimotuzumab alone (p<0.0001). Mice bearing Hep2 human laryngeal tumors had a higher survival rate when treated with intratumoral MeV and/or nimotuzumab (6/10) then those untreated (2/10), and the survival rate of the group treated with MeV and nimotuzumab was higher compared to the groups receiving single treatment (125).

### Lung Cancer

Samuel Ariad reported on the presence of MeV antigens in a large proportion of NSCLC biopsies, contributing to the hypothesis of a potential association between MeV and lung cancer development (126). Based on these results, his team initiated a study investigating the therapeutic potential of a live attenuated MeV (Attenuvax^®^, Merck) delivered subcutaneously as consolidation therapy to stage IIIB/IV, MeV-positive NSCLC patients that are in remission after receiving standard chemotherapy with cisplatin and vinorelbine (NCT00828022). The status of the trial is unknown since 2009.

Zhao et al. preclinically evaluated the anti-tumor effects of the Chinese live attenuated MeV vaccine (based on Hu-191 strain, Chengdu Company of Biological Products) in an immunocompetent mouse model for lung cancer (LCC). Mice were injected intratumorally once every other day for 10 days. The vaccine suppressed tumor growth and significantly prolonged the survival time of tumor-bearing animals compared to control groups. Histological examination revealed that the anti-tumor effects may result from increased induction of apoptosis, tumor necrosis and elevated lymphocyte infiltration (127).

Qi et al. examined the usefulness of the MeV in the prevention of MeV-hemagglutinin (H) protein-expressing tumor growth *in vivo*, and the prolongation of survival of mice injected with MeV vaccine. For the first experiment, mice were vaccinated intramuscularly with MeV vaccine, and 3 weeks later inoculated with MeV-H protein-expressing lung cancer (LCC) and melanoma (B16) cells. For the second, mice were inoculated with H protein-expressing lung cancer and melanoma cells and 7 days later, vaccinated intramuscularly with the MeV vaccine. Active vaccination using MeV vaccine not only protected mice from developing tumors, but also eradicated established tumors. In addition, measles vaccine decreased the population of myeloid derived suppressor cells and regulatory T cells (128).

### Ovarian Cancer

Myers et al. evaluated the *in vitro* and *in vivo* antineoplastic activities of the live attenuated MeV vaccine Attenuvax^®^ (Moraten strain, Merck). In human ovarian cancer cells (SKOV3ip.1, OV202, OV207), the Moraten measles strain caused intercellular fusion and cell death six days post-infection. In an orthotopic intraperitoneal ovarian cancer model, six intraperitoneal administrations of the Moraten vaccine, starting 7 days after tumor implantation, significantly extended survival of all mice compared to control groups. Use of the Attenuvax^®^ vaccine as such in future clinical trials might be complicated as the dose used in these experiments was a thousand times higher than the dose contained in the vaccine. A different formulation or preparation might be required (129).

### Prostate Cancer

Son et al. tested MeV and mumps virus (both purified from Priorix^®^, GSK) in various cancer cell lines of human solid malignancies (kidney, prostate, breast, gastric, liver, lung, cervix, colon, human umbilical cord fibroblast, esophageal, nasopharyngeal and esophageal). When both viruses were used in combination, most tumor cell lines were killed more efficiently (than with either one of the viruses alone) and a stronger oncolytic effect was observed in 11 of the 16 cancer cell lines. Normal cells were not affected. Son et al. also treated mice bearing human prostate (PC-3) tumors twice a week for 3 weeks with single or multiple intratumoral injections of MeV, mumps virus or the combination of both viruses. Both single and multiple injections of the combination of both viruses resulted in significantly decreased tumor volumes and improved survival compared to single injection of either virus. Consequently, they inoculated mice with PC-3 cells pre-treated with the viruses (either alone or in combination) and evaluated response after 24 days. Tumors had disappeared completely in all mice inoculated with cancer cells pre-treated with both vaccines (6/6), compared to control mice (0/6), mice treated with MeV alone (4/6) and mice treated with mumps virus alone (4/6) (130).

## Mumps Vaccine

Since the 1960s, also the mumps vaccine, based on live, attenuated viral strains, is used effectively in prevention of mumps disease. It’s used most in combination vaccines like the measles–mumps–rubella (MMR) vaccine. Building on previous reports of an oncolytic effect of mumps Urabe strain virus in over 290 patients with different malignancies in Japan (131–133), Myers et al. evaluated the *in vitro* and *in vivo* antineoplastic activities of the live attenuated mumps vaccine Mumpsvax^®^ (Jeryl-Lynn strain, Merck). In human ovarian cancer cells (SKOV3ip.1, OV202, OV207), the Jeryl-Lynn mumps strain caused intercellular fusion and cell death six days post-infection. In an orthotopic intraperitoneal ovarian cancer model, one intraperitoneal administration of the Jeryl-Lynn vaccine significantly extended survival of treated mice compared to control groups. The median survival time of mice treated with this mumps vaccine was comparable to that of mice treated with the Moraten MeV described above (129).

Though the dose used in these experiments was a thousand times higher than the dose contained in the vaccine, it remains 10 to 100 times lower than the dose of the Urabe strain used in the Japanese patients. Toxicity reported in these patients was minimal despite the use of a high dose of a strain sometimes responsible for neurotoxicity, which is not the case with the Jeryl-Lynn vaccine. However, using the Jeryl-Lynn vaccine as such in trials will be complicated by the need of a much higher dose and by the fact that Mumpsvax^®^ is not marketed anymore since the Jeryl-Lynn strain has been incorporated in the MMR^®^ vaccine.

Overall, there is limited evidence to support use of the mumps vaccine alone. Additional research on the combination of the mumps and the measles vaccine is described in the measles vaccine section above. A tendency towards synergism between these two *Paramyxoviridae* viruses has been observed in prostate cancer and AML (123, 130).

## Pertussis Vaccine

The best way to prevent whooping cough or pertussis is through immunization. Two main vaccine types are the acellular (Ac) vaccine, containing purified components of *Bordetella pertussis* and developed in the 1980s, and the whole-cell (Wc) vaccine, which is a suspension of the entire though inactivated *B. pertussis* organism developed in the 1930s. Tran et al. reported the case of a 44-year-old male with stage IIIB malignant melanoma (right tibia area). The patient underwent complete excision followed by one year of high-dose interferon-α but developed multiple in-transit metastases during treatment (treated with palliative radiotherapy) and later multiple lesions (skin chest, lung, axillary) six months after completion of the interferon treatment. Three months later, the patient received the tetanus–diphtheria–pertussis vaccine Adacel^®^ (Sanofi Pasteur) for preventive measures after which he developed a local and systemic febrile reaction lasting two days. Five months later, all but one of the nodules had vanished (82).

Although no mechanism for spontaneous regression of melanoma has been established, the authors referred to the hypothesis of Krone et al. about immune surveillance induced or enhanced by prior contacts with pathogens. This is based on an immune-mediated cross-reactivity between homologous peptides being, on the one hand, the human endogenous retroviruses (herv) like the herv-k-mel viral marker prevalent in melanomas, and on the other hand, the peptides in human pathogens (134). Even though testing for herv-k-mel in their patient’s samples was not available, Tran et al. suggested that exposure to the tetanus–diphtheria–pertussis vaccine might have induced a cross-reactivity against melanoma by cytotoxic T lymphocytes (82).

Yano et al. examined the preclinical activity of two pertussis vaccines as adjuvants for the induction of anti-tumor activities. The authors showed through several experiments that both pertussis vaccines were capable of stimulating T helper (Th) cell activities, although the Ac vaccine was less potent in inducing Th1 activity than the Wc vaccine. Anti-tumor responses were observed in two lymphoma mice models (using EL4 and EG7 cells) immunized with a model tumor antigen (OVA-I peptide) with or without the pertussis vaccine before and after intradermal inoculation with tumor cells. When Ac vaccine was added as adjuvant, tumor growth became slower but the Wc vaccine was able to more strongly suppress tumor growth. Similar responses were seen in mice immunized with an endogenous tumor antigen [Wilms’ tumor 1 (WT1) gene product]. Almost all mice receiving the Wc vaccine as adjuvant survived after injection with a lethal dose of the leukemia FBL3 cells, while immunization with an endogenous tumor antigen and the Ac vaccine were less protective. Investigating the long-term effects of WT1 peptide immunization with the Wc vaccine, the authors concluded the Wc vaccine doesn’t induce auto-immune responses and that repeated administration of the Wc vaccine together with a tumor peptide is therefore considered to be relatively safe (135).

## Pneumococcus vaccine

For more than 30 years, vaccines have been used to prevent pneumococcal disease caused by *Streptococcus pneumoniae*. Both conjugate vaccines and polysaccharide vaccines exist target multiple prevalent serotypes. Noonan et al. performed a phase 2 study to determine whether the immunomodulatory drug lenalidomide combined with a pneumococcal vaccine (Prevnar 7^®^, Pfizer) could elicit tumor-specific immune responses in relapsed multiple myeloma patients (NCT00445484). Patients received lenalidomide (25 mg/d on days 1 to 21 of each 28-day cycle) for six cycles in combination with one of two randomly assigned vaccine schedules: cohort A received their first vaccine two weeks before starting lenalidomide and their second on day 14 of cycle 2, and cohort B received their first vaccine on day 14 of cycle 2 and their second on day 14 of cycle 4. Overall response rates were 10% (1/10 patients) in cohort A and 57% (4/7 patients) in cohort B. The tumor-specific immune response increased in cohort B with an average antigen-specific CD3 cell percentage of 7.7% up from a baseline of 2.25% (P=0.003). Cohort A showed no significant induction of a tumor-specific response (20).

The work of Noonan et al. led to additional research on the pneumococcus vaccine and lenalidomide treatment. An ongoing phase 2 study in early-stage asymptomatic chronic lymphocytic leukemia and small lymphocytic lymphoma patients is testing whether 2 doses of a pneumococcal vaccine (Pneumovax 23^®^, Merck Sharp & Dohme) administered either concurrently or sequentially to low-dose lenalidomide can improve both the immune and clinical responses against these diseases (NCT01351896). A phase 2 study assessing progression-free survival in myeloma patients with (near) complete remission receiving lenalidomide alone or with Prevnar 13^®^ and/or an allogeneic myeloma vaccine, is recruiting (NCT03376477).

A pilot study evaluating the safety and feasibility of an intratumoral injection of unmodified autologous DCs and a pneumococcal vaccine (Prevnar 13^®^, Pfizer) after high-dose conformal external beam radiotherapy is recruiting patients with unresectable liver cancer (NCT03942328).

## Rotavirus Vaccine

Oral live, attenuated rotavirus vaccines are available for the prevention of diarrheal rotavirus disease since the 2000s. One article reports the investigation of the effect of the rotavirus vaccine in cancer. Shekarian et al. used intratumoral injection of the rotavirus vaccine as a means to stimulating pattern recognition receptors for the priming of the anti-tumor immunity in mice models of pediatric cancers. Intratumoral injection of the vaccine had significant anti-tumor activity, at least partly due to a direct cytotoxic effect. Cytotoxicity had already been demonstrated by others with various rotavirus strains (136), but was confirmed here with a commercially available vaccine in several cancer lines at doses that were not cytotoxic to normal cells. They then demonstrated that intratumoral injections of the vaccine could overcome resistance and synergize with anti-CTLA-4 and anti-PD-L1 antibodies in four different models (Neuro2a neuroblastoma, A20 B-cell lymphoma, EMT6 breast mammary carcinoma and CT26 colon carcinoma). In a final experiment, the combination of intratumoral rotavirus vaccine and systemic anti-CTLA-4 antibody cured all mice with complete regressions of both injected and non-injected tumors in both the neuroblastoma and the B-cell lymphoma models (11).

Mechanistically, the cancer cell-selective cytotoxicity of intratumoral injection of the rotavirus vaccine leads to type-I interferon induction at the tumor site, with activation of tumor-infiltrating myeloid cells and CD8^+^ T-cells. The synergism observed between intratumoral injection of the rotavirus vaccine and anti-CTLA-4 was mainly CD8^+^ T-cell-dependent, though the B-cell and humoral response was not evaluated.

## Smallpox Vaccine (aka Vaccinia)

The first ever vaccine developed for prevention of an infectious disease was the smallpox vaccine. A global vaccination program in the 1960s and 1970s with this live, attenuated vaccine, led to the eradication of smallpox. Several observational studies (137, 138), but not all (139), have revealed that various infectious diseases and/or immunization with vaccinia, BCG or both in childhood were associated with a reduction in the risk of developing melanoma. Based on these data, Kölmel et al. asked whether immunization with vaccinia and/or BCG during childhood affected long-term survival after surgical removal of a primary melanoma. In a cohort of 542 melanoma patients, a history of vaccination with vaccinia was associated with longer survival (adjusted HR, 0.52; 95% CI, 0.34–0.79). A positive association was also reported between survival and history of BCG vaccination (140).

To explain the mechanism of action, Krone et al. proposed a hypothesis that immune surveillance induced or enhanced by prior contacts with pathogens is unexpectedly cross-reactive to a cellular ‘marker of melanoma risk’. The latter refers to the herv-k-mel-antigen, a product of a pseudo-gene that is closely associated with the env-gene of herv-K, and prevalent in melanoma. A suppressive immune reaction appears to inhibit the expression of the herv-K env-gene, that could otherwise result in malignant transformation years or even decades later as it disturbs the redox-regulation within cells. BCG and vaccinia both have sequence homologies with the herv-k-mel antigen, enabling them to deliver co-stimulatory signals necessary for the induction of the mechanisms protecting against melanoma (134).

Despite the interesting hypothesis, careful interpretation of the data above is required (141) because of the potential residual confounding and the failure of two well-conducted randomized trials of adjuvant vaccination with vaccinia melanoma lysates (142, 143).

Recently, a Canadian phase 1/2 clinical trial (NCT04410874) started recruitment of 45 patients with non-melanoma skin cancers (basal cell carcinoma and squamous cell carcinoma). The Imvamune^®^ vaccinia vaccine will be administered intratumorally twice at one of three doses. Primary outcomes are maximum tolerated dose and objective response rate.

Looking towards other cancer types, Villumsen et al. assessed the effect of BCG and smallpox vaccinations on the subsequent risk of developing malignant lymphoma or leukemia in a Danish population and did not find any association between smallpox vaccination and either leukemia or lymphoma diagnosis (144).

## Tetanus Vaccine

Since its development in 1924, the use of tetanus toxoid containing vaccines has been a very effective strategy to prevent against *Clostridium tetani* bacterial infection. The evidence for the tetanus vaccine in cancer points to a role similar to what has been described for the diphtheria vaccine. With a few exceptions, the majority of the papers report on the use of Td toxoids. It should be noted that a case report of melanoma regression after vaccination with a trivalent diphtheria-tetanus-pertussis vaccine has been described in the pertussis section (82).

Kaufman et al. performed a randomized controlled trial of ALVAC-CEA/B7.1 vaccine, with or without tetanus toxoid adjuvant, and chemotherapy in metastatic colorectal cancer patients (21). ALVAC-CEA/B7.1, an engineered canarypox vaccine expressing CEA and B7.1 (CD80), had previously been tested in a phase I clinical trial that had shown some signs of clinical activity as well as safety and tolerability (145). The rationale for adding tetanus vaccination was to assess whether this would enhance the response to the ALVAC vaccine. Results showed that the combination of chemotherapy and anticancer vaccines was safe and in addition to biomarker responses some patients showed clinically meaningful responses. However, there were no differences between patients receiving ALVAC alone and those that received tetanus vaccine (21).

Mitchell et al. explored the use of Td in combination with an experimental DC vaccine in a small randomized controlled trial in newly diagnosed glioblastoma patients. Patients (n=12) were randomized to DC vaccine site pre-conditioning with unpulsed autologous matured DCs or Td. Patients in the Td group (n=6) showed improved PFS and OS compared to the unpulsed DC group. Biological correlates included enhanced DC migration to vaccine site-draining lymph nodes. Corroborating studies in mice also slowed tumor progression and enhanced DC migration in a CCL3-dependent manner (22). This strategy is currently being investigated in a randomized trial in newly diagnosed glioblastoma multiforme patients (NCT02366728). Investigators have recently summarized the results of their treatment development program obtained so far (146).

More trials involving Td preconditioning were described in the diphtheria section above.

The effect of pre-immunization was also investigated in a mouse model by Tähtinen et al. Mice pre-immunized with tetanus vaccine were challenged with B16.OVA tumors and treated with an experimental peptide-based vaccine containing both tetanus toxoid- and tumor-specific peptides. Treatment significantly slowed tumor growth compared to treatment with the peptide vaccine alone or tetanus vaccine alone. The tetanus vaccine alone had no effect on tumor growth. Additional experiments combining tetanus pre-immunization, peptide vaccine and checkpoint inhibitors, showed significantly improved responses. Analysis showed that the stimulatory effect was mediated by CD4^+^ memory T-cell populations, suggesting that the effect might be exploited clinically by creating peptide-based vaccines which interact with widely used preventive vaccines in human populations (147). Overall, this series of experiments however does not support a therapeutic role of the tetanus vaccine as such.

Tetanus vaccine is included as a treatment arm in a phase 1/2 trial (NCT02737475) for advanced solid tumors, in combination with the checkpoint inhibitor nivolumab and the experimental immunotherapy BMS-986178, an agonistic anti-OX-40 antibody.

## Typhoid Vaccine

End of the 19^th^ century, a first typhoid vaccine preventing typhoid fever caused by *Salmonella Typhi* infection was developed. The more recent injectable typhoid conjugate and unconjugated polysaccharide vaccines and the oral live, attenuated Ty21a vaccines are much safer. Domingos-Pereira et al. tested the preclinical efficacy and safety of intravesical administration of the typhoid vaccine (using the Ty21a strain) in an orthotopic bladder cancer model. Both BCG and typhoid vaccine instillations starting early (1 day) after tumor implantation resulted in long-term survival. Mice surviving more than 150 days underwent a tumor re-challenge without any additional instillation and all mice survived until the end (140 additional days). Typhoid was superior to BCG when instillations started 5 days after tumor implantation with 50% and 10% of the respective mice surviving 150 days. Reassuringly, the Ty21a strain did not persist either in mice or in human bladder cells (148). In another study, they confirmed these results, even at a low dose of Ty21a, and further elucidated the mechanism of action. A single Ty21a instillation increased both T cells and DCs infiltrations in the bladder at numbers only achieved for BCG by multiple instillations. The anticancer effect relied on T cells and DCs (in contrast with BCG), but not on NK cells and neutrophils (149). A phase I trial of intravesical instillations of Vivotif^®^ (PaxVax Berna) has been initiated based on these findings (NCT03421236).

In contrast to this, in their work focusing on the intratumoral injection of the rotavirus vaccine, Shekarian et al. did not find any single-agent activity of intratumoral injection of the typhoid vaccine in a neuroblastoma model resistant to anti-CTLA-4, anti-PD-1, anti-PD-L1, or their combinations (11). Also, one case-control study reported a positive association between typhoid immunization and the incidence of liver cancer (OR = 6.3, 95% CI: 1.1-37.4) but this association should be questioned by the small number of cases (n=11) and the multiple associations being tested (245 associations tested) (150).

Related to cell-based cancer immunotherapy, Schreibelt et al. explored the role of infectious diseases vaccines in *ex vivo-*generated human monocyte-derived DC maturation. BCG (Nederlands Vaccin Instituut) and Act-HIB^®^ (Aventis Pasteur), a vaccine against *Hemophilus influenzae*, were capable of activating Toll-like receptors (TLR) on DCs, which induced DC maturation and cytokine secretion. The flu vaccine (Influvac, Solvay Pharmaceuticals) and typhoid vaccine (Typhim, Sanofi Pasteur MSD) also generated mature DCs but no extracellular TLR ligands could be found, indicating they might engage with other DC receptors or activate intracellular TLRs. They also compared different combinations of vaccines, as such, and showed that stimulation with a cocktail of BCG, Influvac, and Typhim (all 4% vol/vol) plus prostaglandin E2 (PGE2) gave rise to fully mature DCs that produced large amounts of IL-12p70, suggesting that these 3 vaccines have a synergistic effect on DC maturation that could be useful to improve immunologic and clinical responses in DC vaccination of cancer patients (38).

Based on these *ex vivo* data, Bol et al. performed a clinical trial to study the safety and efficacy of DC therapy in 28 stage III and IV melanoma patients. A prophylactic vaccine cocktail, consisting of BCG, Typhim, and Act-HIB, together with PGE_2_ was used to mature the DCs, that were then injected either combined intravenous/intradermal or intranodal. Tumor antigen-specific immune responses were observed in almost all patients, and, despite the small sample size, were correlated with survival of human leukocyte antigen A*02:01-positive stage IV patients, with an overall survival ranging from 14 to 28 months in patients with tumor-associated antigen-specific T cells (n = 4), whereas in the absence of these cells (n = 7) the overall survival ranges from 3 to 11 months (p = 0.003). Despite the functional tumor-specific responses, vaccination induced local and systemic grade 2 and 3 toxicity. In the authors’ opinion, the side effects in this study were of a higher grade and occurred earlier and at a faster rate than is usually seen in DC vaccination. Toxicity is most likely caused by the BCG vaccine in the maturation cocktail, according to the authors, because the use of BCG is known to induce the side effects, they saw: flu-like symptoms, fever and lymphadenopathy and erythema of the overlying skin. The authors also hypothesize that this DC vaccine trapped in the lungs after intravenous injection attract BCG-specific immune cells, thereby causing pneumonitis. It was concluded that toxicity precludes general application of this DC therapy (23).

## Varicella-Zoster Vaccine

Varicella-zoster virus (VZV) causes varicella (chickenpox) and herpes zoster (shingles). Varicella-Zoster vaccines, available since 1980s, contain live, attenuated VZV (Oka strain). No data on direct or indirect anti-tumoral effects with the Varicella-Zoster vaccine were found. However, one trial uses the vaccine to help expand Chimeric Antigen Receptor (CAR) T-cells targeting GD-2, a disialoganglioside expressed by neuroblastoma and other cancers (NCT01953900). The trial is a safety trial in patients with refractory osteosarcoma or neuroblastoma. The investigators have developed VZV-specific T cells expressing a CAR for GD2 and use the VZV to stimulate *in vivo* the expansion of those CAR T-cells (151).

## Yellow Fever Vaccine

Live, attenuated viral vaccines are used for immunization against yellow fever in high risk and endemic areas since the late 1930s. Aznar et al. used *in vitro* data to show that yellow fever (YF) vaccine (live 17D strain) kills both mouse and human cancer cells (human tumor cell lines representing colon cancer, renal cell carcinoma, breast cancer, and melanoma). Non-transformed fibroblasts were not affected. Intratumoral injection of 17D in female C57BL/6 bearing established MC38 colon carcinoma and B16-OVA melanoma tumors showed delay in tumor growth, improved survival but no cures. Using the MC38 model contralateral tumors not injected also trended to slowed tumor growth without showing evidence of viral replication in the non-injected tumors. Analysis showed that tumor growth retardation was mediated *via* CD8^+^ T cells. A clear synergy was shown when used with anti-CD137 but not anti-PD-1 antibodies. Further testing indicated that pre-immunization with 17D to promote YF immunity prior to tumor cell inoculation improved efficacy of intratumoral treatment once tumors had become established, as did earlier treatment (6 days after inoculation rather than 8 days) (152).

Some support for the finding that immunization with 17D might have preventive effects is provided by an observational study by Mastrangelo et al. They showed that in a cohort of 12,804 Italian women vaccinated with YF 17D vaccine, breast cancer risk was reduced by about 50% (incidence rate ratio=0.46; 95% CI=0.26–0.83) 2 years after vaccination (153). An earlier study by some of the same authors had investigated the effects of YF 17D immunization on melanoma risk. This cohort study, including a nested case-controlled study of melanoma and other cancers, showed that YF immunization was associated with a reduced risk of melanoma after 10 years (OR=0.26, 95% CI=0.07–0.96) (154). However, a subsequent study in US military personnel by Hodges-Vazquez et al. reported a no significant protective effect (OR=0.93, 95% CI=0.78–1.10) (155). The Mastrangelo study reported a protective effect after 10 years, whereas the US military study assessed the period *up to* 10 years. Importantly, one case-control study reported a positive association between YF immunization and the incidence of liver cancer (OR = 8.7, 95% CI: 1.0-76.3) but this association should be questioned by the small number of cases (n=11) and the multiple associations being tested (245 associations tested) (150).

A number of studies have shown that YF 17D induces polyvalent immune responses *via* activation of multiple TLRs on DC populations thereby eliciting a broad spectrum of innate and adaptive responses (156, 157).

To date the anticancer effects of YF 17D have not been investigated in a clinical trial setting.

## Discussion

Of the initial list of 31 infectious diseases for which a preventive vaccine exists, we found data supporting a possible therapeutic role in oncology for 16 of them. They are being investigated in a broad range of cancer types and in several administration routes (intratumoral, intravesical, in addition to their traditional mode of administration, and mostly intramuscular). In **Table 2**, we summarize potential next steps for each individual vaccine repurposing strategy.

For 10 vaccines (BCG, diphtheria, tetanus, HPV, influenza, measles, pneumococcus, smallpox, typhoid and varicella-zoster), clinical trials have been conducted or are ongoing. Within the remaining 6, strong preclinical evidence supports further evaluation of the rotavirus and yellow fever vaccine in carefully designed clinical trials. We believe that for the cholera vaccine in colorectal cancer, more preclinical data on efficacy and mode of action is needed before clinical translation. For the pertussis vaccine, preclinical research should explore its potential anticancer effect in melanoma, based on a case report, and further characterize the increased immune response in lymphoma. Data is less convincing for the hepatitis B vaccine. Finally, data in favor of the mumps vaccine alone is very limited though combining it with the measles vaccine might be a better strategy for future research.

All the vaccines we have identified are intended to induce or improve anti-tumor (immune) responses. However, practical aspects and mechanisms of action are very different from one vaccine to another and from trial to trial. Some vaccines may be effective as single agents (typhoid, measles, yellow fever vaccine) while others are considered in combination with existing or experimental immunotherapies (Td, influenza, pneumococcus and rotavirus vaccine). Sequence in these combinations and frequency of administration are variable and often neglected in trial designs and research experiments, though of high importance (5). A remarkable setting is the perioperative use of the influenza vaccine to limit or prevent the NK cell dysfunction induced by cancer surgery (9). Pre-existing immunity against the vaccine pathogen may be required for some vaccines to be effective (4, 152,), but may hamper the efficacy of the vaccines when a direct oncolytic effect is sought (158). Several methods to overcome the hurdle of acquired immunity and neutralizing antibodies are being explored, like the use of DC carriers (159), cyclophosphamide immunosuppression (160), repeat oral administration (161), etcetera.

Some of the most striking clinical findings are coming from the therapeutic use of the HPV and the measles vaccine in cutaneous tumors (SCC of the skin for HPV, cutaneous T-cell lymphomas for measles). While both tumors are not considered as major therapeutic challenges, mainly because they already have several effective therapeutic options, the preliminary results are encouraging and will hopefully lead to confirmatory trials. This may be particularly relevant for HPV in therapeutically challenging cases of SCC of the skin.

Another striking finding was the superiority of intravesical typhoid vaccine instillations over BCG instillations in a preclinical model of bladder cancer, which is now the subject of a phase I trial. Improving the efficacy of immunotherapeutic intravesical instillations would be a great advance in the treatment of the 5^th^ most frequent, but rather neglected, cancer (149).

Compared to previous review articles (4, 8, 12), we have used a well-defined methodology to capture articles from the literature (18). Our review also intentionally focused on articles reporting the use of the unmodified infectious diseases vaccines. We do not dispute the scientific justification of modifying existing vaccines (or using them as vectors) to create new, more potent, anticancer therapies. However, any modification should be balanced against the odds of subsequent clinical translation. Any modification will have consequences in terms of production costs, Good Manufacturing Practices manufacturing and regulatory requirements to conduct clinical trials. Ultimately, an adequate business model will need to be built to pursue the clinical evaluation of the new products. A clinical development using an existing product is simpler though not necessarily straightforward (18).

Our review has some limitations. We limited our search to articles published from 2000 on. We may also still have missed important findings, despite comprehensive literature queries.

Because our review has a clinical development focus, we have not reported detailed information about the biological and immunological mechanisms of action of the different vaccines and their use in cancer treatment. What becomes clear from this review is that there are major differences in the direct oncolytic and indirect immune modulation mechanisms of action between vaccines and within vaccines. Combinations and sequence of treatments, and mode of administration play an important role as well and deserve more attention in future research. The clinical development of each vaccine strategy has its own rationale and improving the mechanistic understanding of the efficacy of the vaccine should be integrated into each development program.

In conclusion, we have found that multiple infectious disease vaccines are currently being explored in an unmodified form, both preclinically and clinically, in oncology. In order to inform future research work to further develop promising vaccine repurposing strategies, a proposal for next research steps is made. The repurposing of existing vaccines may represent a cost-effective strategy to bring new therapeutic options to patients but requires special care in terms of clinical development and regulatory requirements.

## Author Contributions

LV and GB defined the scope of the review. PP ran the queries and prepared the databases for off-line processing. LV and GB screened the abstracts and trials for relevance. LV, PP, and GB wrote the manuscript. PP and AV reviewed the manuscript and provided input with the interpretation. All authors contributed to the article and approved the submitted version.

## Funding

All authors are employees of the Anticancer Fund.

## Conflict of Interest

The authors declare that the research was conducted in the absence of any commercial or financial relationships that could be construed as a potential conflict of interest.
